# Effectiveness of the new integrated strategy to control the transmission of *Schistosoma japonicum* in China: a systematic review and meta-analysis

**DOI:** 10.1051/parasite/2018058

**Published:** 2018-11-16

**Authors:** Chunyan Qian, Yuefeng Zhang, Xinyan Zhang, Chao Yuan, Zhichao Gao, Hong Yuan, Jiang Zhong

**Affiliations:** 1 Yuhang Branch, The Second Affiliated Hospital of Zhejiang University Hangzhou 311100 Zhejiang Province PR China; 2 School of Life Sciences, Fudan University Shanghai 200433 PR China; 3 Department of Clinical Laboratory, The Obstetrics and Gynecology Hospital of Fudan University Shanghai 200001 PR China; 4 Shanghai Skin Disease Hospital Shanghai 200443 PR China

**Keywords:** *Schistosoma japonicum*, integrated strategy, morbidity control, effectiveness evaluation, systematic review, meta-analysis

## Abstract

Since 2004, the national schistosomiasis control strategy in China has shifted from the morbidity control strategy (conventional strategy) to an integrated strategy (new strategy). We investigated the effectiveness of the new strategy and compared it against the conventional strategy. We retrieved from electronic databases the literature regarding the new strategy published from 2000 to 2017. The effect of the new or conventional strategy on infection by *Schistosoma japonicum* of humans and snails (*Oncomelania hupensis*) was evaluated with pooled log relative risk (logRR). A total of only eight eligible publications were included in the final meta-analysis. The results showed that implementation of the new strategy reduced the infection risk by 3–4 times relative to the conventional strategy. More specifically, the conventional strategy caused a reduction in both human (logRR = 0.56, 95% CI: 0.12–0.99) and snail infections (logRR = 0.34, 95% CI: −0.69–1.37), while the new strategy also significantly reduced both human (logRR = 1.89, 95% CI: 1.33–2.46) and snail infections (logRR = 1.61, 95% CI: 1.06–2.15). In contrast to the conventional strategy, the new strategy appeared more effective to control both human (logRR difference = 1.32, 95% CI: 0.78–1.86) and snail infections (logRR difference = 1.53, 95% CI: 0.76–2.31). Our data demonstrate that the new integrated strategy is highly effective to control the transmission of *S. japonicum* in China, and this strategy is recommended for schistosomiasis elimination in other affected regions across the world, with adaptation to local conditions.

## Abstract (fr)

Depuis 2004, la stratégie nationale de lutte contre la schistosomiase en Chine est passée d’une stratégie de contrôle de la morbidité (stratégie conventionnelle) à une stratégie intégrée (nouvelle stratégie). Nous avons examiné l’efficacité de la nouvelle stratégie et l’avons comparée à la stratégie conventionnelle. Nous avons extrait des bases de données électroniques la littérature concernant la nouvelle stratégie publiée de 2000 à 2017. L’effet de la stratégie nouvelle ou conventionnelle sur l’infection par *Schistosoma japonicum* des humains et des mollusques (*Oncomelania hupensis*) a été évalué avec le risque relatif de log groupé (logRR). Au total, seulement 8 publications éligibles ont été incluses dans la méta-analyse finale. Les résultats ont montré que la mise en œuvre de la nouvelle stratégie réduisait de 3 à 4 fois le risque d’infection par rapport à la stratégie conventionnelle. Plus spécifiquement, la stratégie conventionnelle a entraîné une réduction des infections humaines (logRR = 0.56, IC à 95 % : 0.12–0.99) et des infections des mollusques (logRR = 0.34, IC à 95 % : −0.69–1.37), tandis que la nouvelle stratégie a aussi réduit les infections humaines (logRR = 1.89, IC à 95 % : 1.33 à 2.46) et des mollusques (logRR = 1.61, IC à 95 % : 1.06 à 2.15). Contrairement à la stratégie conventionnelle, la nouvelle stratégie a semblé plus efficace pour contrôler à la fois les infections humaines (différence de logRR = 1.32, IC à 95 % : 0.78–1.86) et les infections des mollusques (différence de logRR = 1.53, IC à 95 % : 0.76–2.31). Nos données démontrent que la nouvelle stratégie intégrée est très efficace pour contrôler la transmission de *S. japonicum* en Chine et que cette stratégie est recommandée pour l’élimination de la schistosomiase dans d’autres régions touchées du monde, avec une adaptation aux conditions locales.

## Introduction

Schistosomiasis is a parasitic disease caused by the blood flukes of the genus *Schistosoma* [8]. It ranks second after malaria among the global human parasitic diseases in terms of socio-economic and public health importance in tropical and subtropical areas [8]. Worldwide, this neglected tropical disease affects more than 207 million people in 78 countries, with 779 million people at risk of infection [37], leading to 0.2 million deaths [29] and 1.75–2.00 million disability adjusted life years (DALYs) each year [18].

Three major *Schistosoma* species are known to infect humans, including *S. haematobium*, *S. mansoni*, and *S. japonicum* [8]. Schistosomiasis japonica, caused by infection with the parasite *S. japonicum*, is endemic mainly in China, the Philippines, and parts of Indonesia [8]. Concerted control efforts since the 1950s have dramatically reduced the number of infections as well as the burden of the disease in the endemic areas of China [10, 40, 62]. However, schistosomiasis japonica remains a major public health concern in China, as one of the four priorities for communicable disease control defined by the central government [44]. Currently, the disease remains endemic in the marshland and lake regions of five provinces along the middle and lower reaches of the Yangtze River, and in some mountainous areas in the provinces of Sichuan and Yunnan, and over 0.7 million people living in China are thought to have the disease [63].

The national strategy for schistosomiasis control has shifted three times in China since it was first initiated: transmission control strategy (from mid-1950s to early 1980s), morbidity control strategy (from mid-1980s to 2003), and the new integrated strategy (2004 to present) [53, 54]. The morbidity control strategy, also known as the conventional strategy, focuses on synchronous chemotherapy for humans and bovines [4], and the new strategy developed in 2004 intervenes in the transmission pathway of schistosomiasis japonica, mainly including replacement of bovines with machines, prohibition of grazing cattle in the grasslands, improving sanitation, installation of fecal-matter containers on boats, praziquantel drug therapy, snail control, and health education [42]. This new integrated control strategy has proven to be highly effective to reducing the rate of *S. japonicum* infection in both humans and the intermediate host snails [24, 26, 39, 43, 46, 65, 66]. However, the effectiveness of this new integrated strategy varies in previous reports in terms of the implementation in different endemic regions and different local circumstances [36]. We therefore present a systematic literature review and meta-analysis to evaluate the effectiveness of the new integrated strategy to control the transmission of *S. japonicum* in China, and compare results against those of the conventional strategy.

## Materials and methods

### Search strategy and data source

The studies pertaining to the effectiveness of the new strategy for schistosomiasis control that were published during the period from January 1st, 2000 through December 31th, 2017, were jointly searched in electronic databases, including PubMed, Web of Science, Embase, Proquest, Cochrane Library, China National Knowledge Infrastructure (CNKI), the Wanfang Database and VIP Database. The terms we used included “schistosomiasis”, in combination with “integrated control strategy”, “comprehensive control strategy” or “infectious source control measures”. The title and abstract of each publication screened were read carefully, and the full texts were reviewed.

### Study selection

Both inclusion and exclusion criteria were defined for identifying the publications included in our meta-analysis. Inclusion criteria involved: (1) the control measures targeting schistosomiasis japonica; (2) the implementation of the study in China; (3) a detailed description of integrated control interventions with emphasis on control of infectious source of schistosomiasis; (4) inclusion of both study and control areas, and assessment of effectiveness in both groups; (5) a description and evaluation of prevalence of human *S. japonicum* infection and snail infection as outcomes of the interventions; and (6) available full text for review. The literature articles that met the following criteria were excluded: (1) lack of control areas or lack of effectiveness evaluation in control areas; (2) no description of quantitative outcomes of interventions; (3) the original data regarding the outcomes of interventions were not available; and (4) the full text was unavailable.

### Assessment of publication bias

A funnel plot was drawn to evaluate literature quality. We tested funnel plot asymmetry based on the linear regression method [38] using the metabias function in the meta package of *R* software [34]. We used a cut-off *p*-value of <0.05 to determine the asymmetry of the funnel plot, and further the presence of publication bias.

### Meta-analysis

We carried out a meta-analysis (fixed- or random-effects models) using the RMA function in the metafor package of *R* software [41]. The effects of the new or conventional strategy in human/snail studies were evaluated with pooled log relative risk (logRR) and the corresponding 95% confidential interval (CI). We then calculated the logRR difference between the strategies and the standard error (*SE*) as below:

logRR difference=logRR new strategy-logRR conventional strategy,

$$\mathrm{SE}\left(\mathrm{logRR}\;\mathrm{difference}\right)=\sqrt{\mathrm{SE}{\left(\mathrm{logRR}\;\mathrm{new}\;\mathrm{strategy}\right)}^{2}+\mathrm{SE}{\left(\mathrm{logRR}\;\mathrm{conventional}\;\mathrm{strategy}\right)}^{2},}$$

from which we further compared the two strategies with pooled logRR differences. In all analyses, Cochran’s *Q* test and *I* ^2^ statistics were employed to measure the heterogeneity between studies. A random effects model was employed to estimate overall studies if heterogeneity existed in the data source. Otherwise, a fixed-effect model was reported.

All statistical analyses were performed using *R* software, and a *p*-value of <0.05 was considered statistically significant.

## Results

### Literature searched

**Figure 1.**
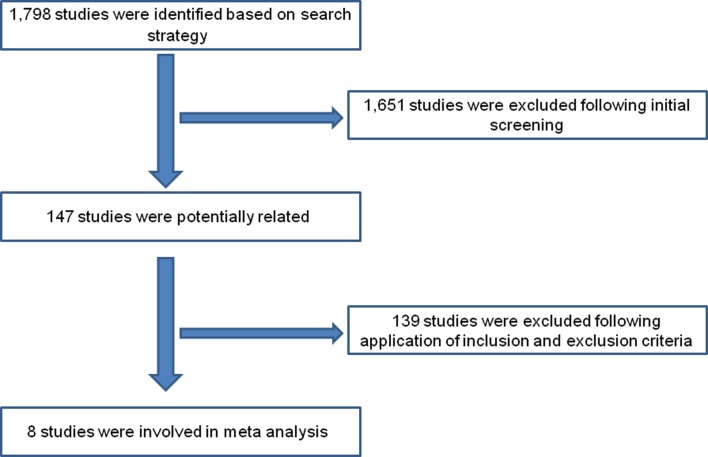
Flowchart of study selection.

A total of 1798 publications were identified, of which 147 articles were potentially relevant according to the initial screening. Following the application of the inclusion and exclusion criteria, 139 studies were excluded. Finally, eight papers that examined the effectiveness of the new strategy were included in the meta-analysis (Fig. 1), of which five included two study areas and two control areas. Table 1 describes the general characteristics of the studies enrolled in the analysis [15, 25, 27, 42, 43, 59–61].

**Table 1.** Characteristics of the studies included in the meta-analysis

No.	Study region	Study period	Integrated interventions targeting control of infectious sources	Study measurements	References
1	Anhui province	2002–2003	Replacement of cattle with machines, improvement of sanitation, and building lavatories and latrines	Human *S. japonicum* infection and snail infection	[61]
2	Mountainous regions of Yunnan province	2006–2007	Improvement of sanitation, and building lavatories and latrines and prohibition of grazing cattle in the grasslands	Human *S. japonicum* infection and snail infection	[27]
3	Poyang Lake region	2005–2007	Removing cattle from snail-infested grasslands, providing farmers with mechanized farm equipment, improving sanitation by supplying tap water and building lavatories and latrines, providing boats with fecal matter containers, and implementing an intensive health education program	Human *S. japonicum* infection and snail infection	[42]
4	Four provinces of Anhui, Hubei, Hunan and Jiangxi	2005–2008	Removing cattle from snail-infested grasslands, providing farmers with mechanized farm equipment, improving sanitation by supplying tap water and building lavatories and latrines, providing boats with fecal matter containers, and implementing an intensive health education program	Human *S. japonicum* infection and snail infection	[43]
5	Xuancheng city of Anhui province	2006–2007	Replacement of cattle with machines, improvement of sanitation, and building lavatories and latrines	Human *S. japonicum* infection and snail infection	[59]
6	Jingzhou city of Hubei province	2010–2011	Replacement of cattle with machines, and prohibition of grazing cattle in the grasslands	Human *S. japonicum* infection and snail infection	[25]
7	Gong’an county of Hubei province	2009–2011	Building fences to limit the grazing area for cattle, building safe pastures for grazing, improving the residents’ health conditions and facilities	Human *S. japonicum* infection and snail infection	[15]
8	Jinxian county along Poyang Lake region	2004–2005	Grazing and marshland isolation, replacing cattle with tractors, and improving access to water and sanitation facilities	Human *S. japonicum* infection and snail infection	[60]

### Literature quality

**Figure 2.**
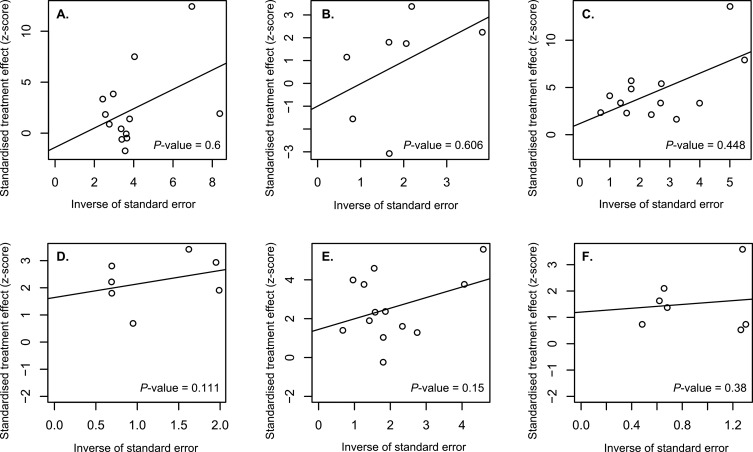
Funnel plot shows asymmetry for the studies included in this analysis. (A) the funnel plot of the studies reporting the effectiveness of the conventional strategy on the control of human *Schistosoma japonicum* infection; (B) the funnel plot of the studies reporting the effectiveness of the conventional strategy on the control of *Oncomelania hupensis* snail infection; (C) the funnel plot of the studies reporting the effectiveness of the new strategy on the control of human *Schistosoma japonicum* infection; (D) the funnel plot of the studies reporting the effectiveness of the new strategy on the control of *Oncomelania hupensis* snail infection; (E) the funnel plot of the studies comparing the effectiveness between the new strategy and the conventional strategy on the control of human *Schistosoma japonicum* infection; (F) the funnel plot of the studies comparing the effectiveness between the new strategy and the conventional strategy on the control of *Oncomelania hupensis* snail infection.

We evaluated the quality of the articles included in this study according to the funnel plot asymmetry using metabias function in the *R* package meta. Symmetry of the funnel plot was observed, with all *p* values of >0.05 (Fig. 2). The results indicated no publication bias present in the articles used in the meta-analysis.

### Meta-analysis

A heterogeneity test revealed the presence of heterogeneity among studies that reported the effect of the conventional strategy on the control of human *S. japonicum* infection (*I* ^2^ = 90.34, *p* < 0.001) and snail infection (*I* ^2^ = 83.52, *p* < 0.001), and the new integrated strategy on the control of human infection (*I* ^2^ = 86.39, *p* < 0.001). No heterogeneity was detected among the studies reporting the alteration of snail infection caused by the new strategy (*I* ^2^ = 0.92, *p* = 0.361). We then estimated pooled logRR and the corresponding 95% CI using random and fixed effects models, respectively.

**Figure 3.**
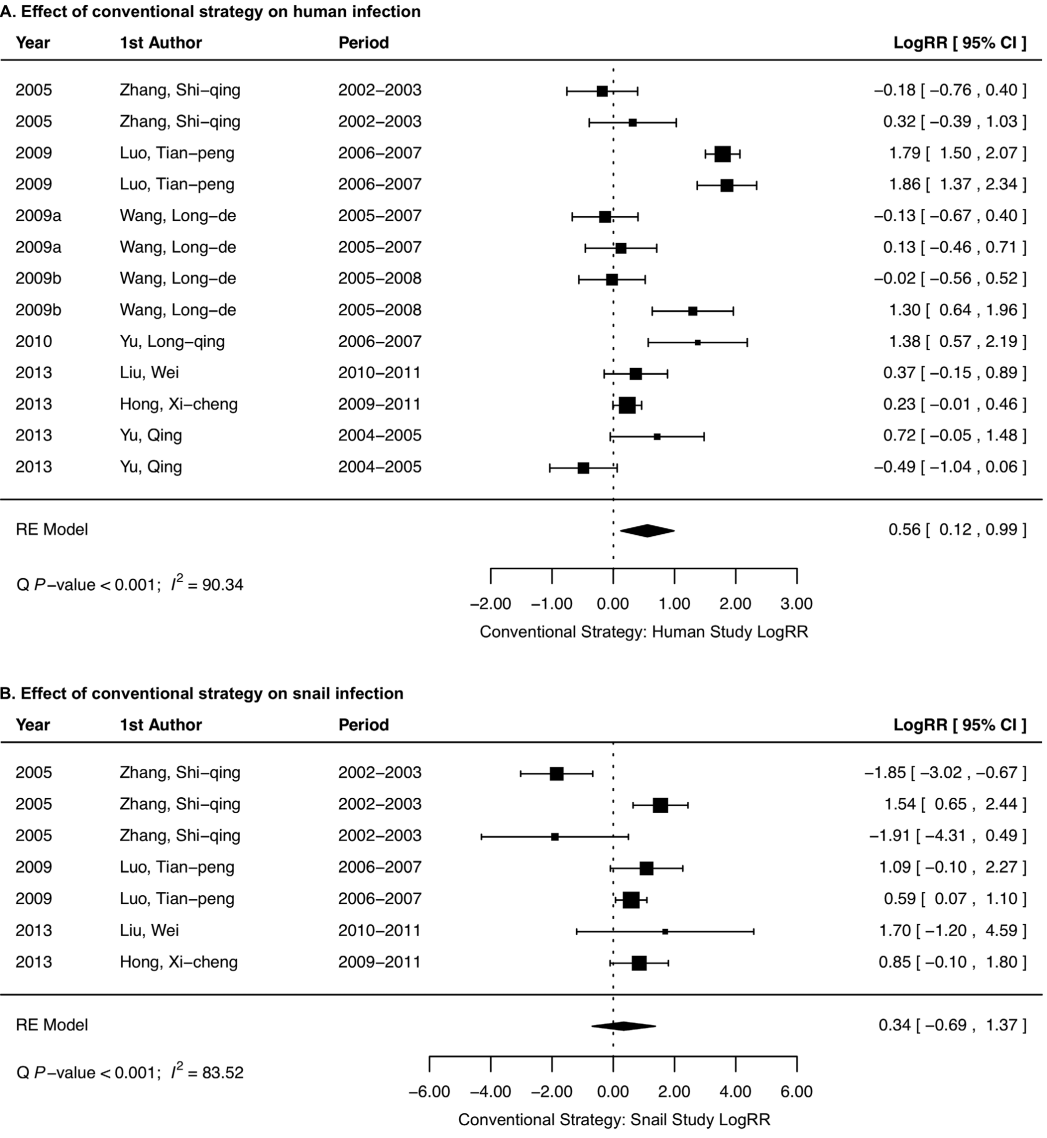
Effectiveness of the conventional strategy on the control of human *Schistosoma japonicum* infection (A) and *Oncomelania hupensis* snail infection (B).

**Figure 4.**
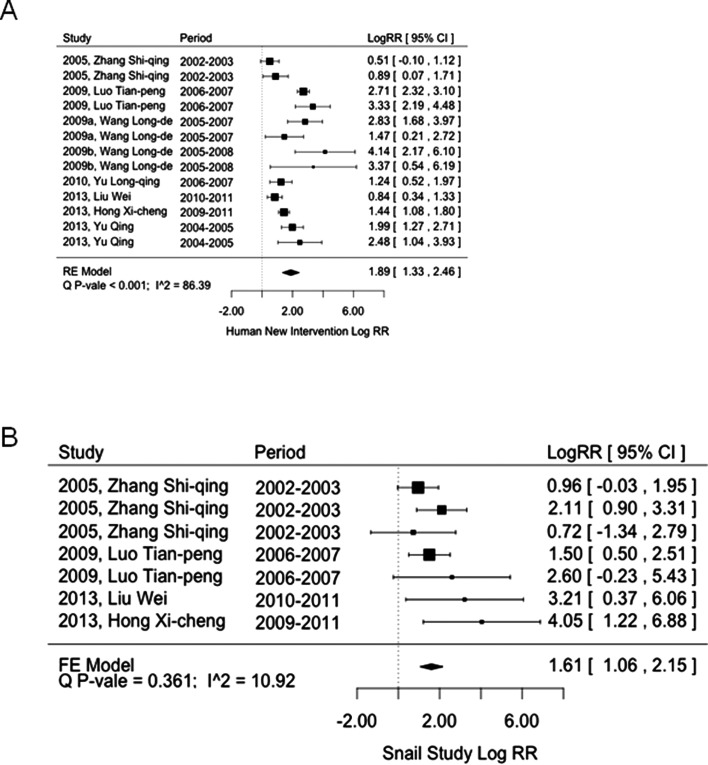
Effectiveness of the new strategy on the control of human *Schistosoma japonicum* infection (A) and *Oncomelania hupensis* snail infection (B).

**Figure 5.**
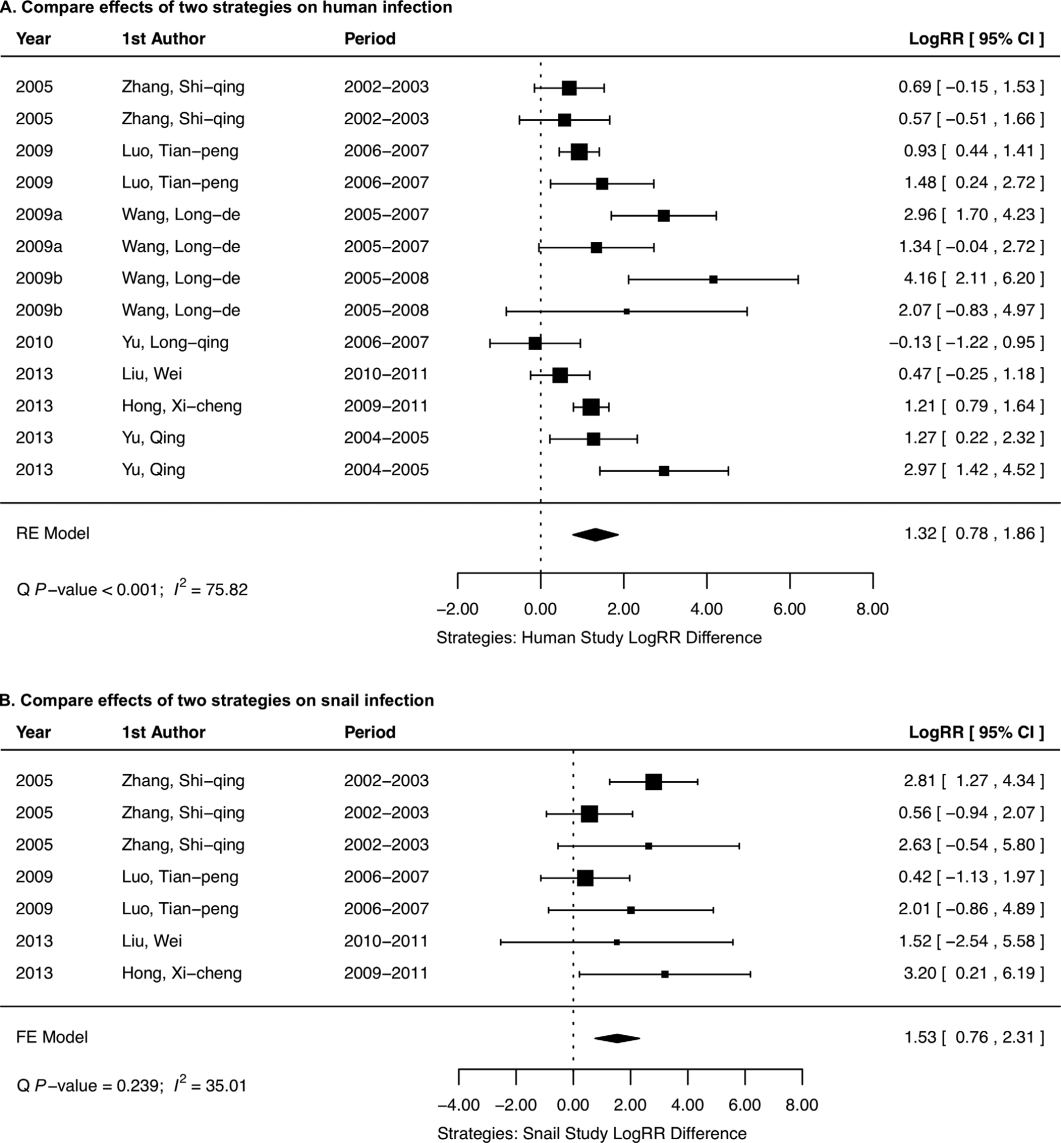
Comparison of the conventional strategy versus the new strategy on the control of human *Schistosoma japonicum* infection (A) and *Oncomelania hupensis* snail infection (B).

We found that the implementation of the conventional strategy caused a reduction in both human *S. japonicum* (logRR = 0.56, 95% CI: 0.12–0.99; Fig. 3A) and snail infections (logRR = 0.34, 95% CI: −0.69–1.37; Fig. 3B), while the new strategy significantly reduced both human *S. japonicum* (logRR = 1.89, 95% CI: 1.33–2.46; Fig. 4A) and snail infections (logRR = 1.61, 95% CI: 1.06–2.15; Fig. 4B). In other words, the conventional strategy reduced the risk of infection by 1.75-fold (95% CI: 1.13–2.69 fold) in humans and 1.4-fold (95% CI: 0.5–3.94 fold) in snails, while the new strategy reduced 6.62-fold (95% CI: 3.78–11.7 fold) the risk of infection in humans and 5-fold (95% CI: 2.89–8.58 fold) in snails. Further comparison between these two strategies indicated that the new strategy was 3.74-fold (95% CI: 2.18–6.42) (logRR difference = 1.32, 95% CI: 0.78–1.86; Fig. 5A) more effective in human infection control and 4.62-fold (95% CI: 2.14–10.07) (logRR difference = 1.53, 95% CI: 0.76–2.31; Fig. 5B) more effective in snail infection control as compared to the conventional strategy.

## Discussion

The description of schistosomiasis in China dates back more than two millennia [64]. Historically, this parasitic disease was called the “god of plagues” by Chairman Mao, the founder of the People’s Republic of China [2, 3]. The disease has caused high social and economic burdens because of its very high rates of morbidity and mortality [56].

The Chinese national schistosomiasis control program was launched in the mid-1950s, and has had three different stages: transmission control strategy, morbidity control strategy, and integrated strategy [53, 54]. In the first stage (from mid-1950s to early 1980s), a transmission control strategy was implemented with emphasis on the control of the intermediate host snails, and mass campaigns were launched to eliminate snail hosts by environmental modification and mollusciciding [13]. During this period, snail habitats were greatly reduced, and the number of schistosomiasis cases decreased [45]. The national schistosomiasis control strategy shifted to morbidity control (from the mid-1980s to 2003) as a response to the advent of the highly effective and low-cost schistosomicide praziquantel [1, 5, 47, 51]. During this stage, five out of the 12 provinces that were endemic for the parasite achieved transmission interruption of schistosomiasis [48]. However, the termination of the World Bank Loan Project for Schistosomiasis Control in 2001 [50] and frequent flooding along the Yangtze River basin [49] resulted in a resurgence of schistosomiasis japonica in China [21, 42, 43]. In order to consolidate the achievements attained and to eliminate schistosomiasis in the country, the Chinese government reinforced the national schistosomiasis control program and prioritized schistosomiasis, together with HIV/AIDS, hepatitis B and tuberculosis in communicable disease control [44]. In addition, a new integrated strategy targeting the transmission pathway of schistosomiasis japonica was proposed to stop environmental contamination with schistosome eggs, which emphasizes replacement of cattle with machines, improvements in sanitation, and fencing of water buffaloes, along with health education, praziquantel-based drug therapy and snail control [42].

The new integrated strategy was designed to reduce the role of cattle and humans as sources of *S. japonicum* infection [42]. It has been highly effective in controlling the transmission of *S. japonicum* in the endemic foci of China [6, 11, 14, 20, 58, 65]. Since the new strategy was implemented in various endemic regions and different combinations of interventions were adopted, the effectiveness of the strategy in reducing infection by *S. japonicum* in humans and the intermediate host snails has been found to vary in previous studies. However, there has been no systematic evaluation of this new strategy to control the transmission of *S. japonicum* in China until now. We therefore carried out a systematic literature review and meta-analysis with the aim of performing a pooled analysis of the effectiveness of the new strategy, and comparing the effectiveness of the new strategy with the conventional strategy in reducing *S. japonicum* infection in both humans and snails.

Our meta-analysis showed that the implementation of the conventional strategy caused a reduction in both human *S. japonicum* infection (logRR = 0.56, 95% CI: 0.12–0.99) and snail infection (logRR = 0.34, 95% CI: –0.69–1.37), suggesting that the praziquantel-based morbidity control strategy is effective in reducing *S. japonicum* infection in humans and snails, while the new strategy remarkably reduced both human *S. japonicum* (logRR = 1.89, 95% CI: 1.33–2.46) and snail infections (logRR = 1.61, 95% CI: 1.06–2.15), indicating that the integrated strategy with emphasis on controlling the source of *S. japonicum* infection is effective in controlling the transmission of *S. japonicum*. However, the new strategy appeared more effective in controlling both human *S. japonicum* (logRR difference = 1.32, 95% CI: 0.78–1.86) and snail infections (logRR difference = 1.53, 95% CI: 0.76–2.31) than the conventional strategy.

The morbidity control strategy mainly involves praziquantel-based drug therapy, snail control, and health education interventions [28]. Nevertheless, praziquantel is ineffective in preventing *S. japonicum* infection and re-infection [23], and it is unlikely to eliminate snails completely in the endemic foci [62]. China’s experiences and lessons from the past three decades of schistosomiasis control have shown that the morbidity control strategy is insufficient to eliminate schistosomiasis in the country [62, 67]. In the Philippines, mass drug administration with praziquantel on its own has proven to be ineffective to control the prevalence of schistosomiasis, the intensity of *S. japonicum* infection, or the morbidity of the disease [17, 31, 32]. Moreover, praziquantel-based deworming alone has been proved ineffective to eliminate schistosomiasis from the African mainland [7, 9, 16, 35]. These findings demonstrate that the sustainable control and elimination of schistosomiasis requires an integrated, multidisciplinary and multi-component strategy [30].

The integrated strategy relies on the fact that cattle have been considered as the major infectious source for the transmission of schistosomiasis in the marshland and lake regions of China [12, 22]. It is therefore assumed that the successful intervention packages piloted in the marshland and lake regions are not fully suitable for the hilly and mountainous environments in the Sichuan and Yunnan provinces of China [36]. Although field studies have shown that this new integrated strategy remains effective to control *S. japonicum* infection in humans and snails in hilly and mountainous endemic foci [26, 27, 57], regionally flexible integrated, intersectoral, and setting-specific control strategies driven by local circumstances and data are needed [36].

The present study has some limitations. First, only eight eligible studies were enrolled in the meta-analysis. A total of 147 potentially relevant literatures were initially identified; however, 139 studies were excluded due to unavailability of original data regarding *S. japonicum* infection in humans and snails in the articles. In addition, most of the studies were published in national journals. Therefore, more randomized controlled trials with a rigorous design to evaluate the effect of the integrated control strategy for schistosomiasis japonica seem justified, and the research outcomes are encouraged to be transferred around the world. Second, no stratified analysis was performed. Since there were only eight studies included in the meta-analysis, we evaluated the effectiveness of the new integrated strategy implemented in endemic foci with various endemic types, and did not assess the endemic type-specific effectiveness. Further systematic evaluations recruiting more trials to evaluate the effectiveness of the new integrated strategy for controlling the transmission of *S. japonicum* in the marshland and lake regions, the mountainous regions and plain regions, respectively, seem justified.

In summary, the results of the present study demonstrate that the new integrated strategy with emphasis on the control of the infectious source is highly effective to control the transmission of *S. japonicum* in China. The elimination of schistosomiasis japonica in the country requires continually effective and extensive implementation of an integrated, intersectoral, and setting-specific control strategy. Currently, China is transferring its expertise in schistosomiasis control to Africa, and the Philippines may also learn much from China’s experiences and lessons [52, 55]. Experiences and lessons from China are important for shaping the schistosomiasis elimination agenda [19]. However, there is still a need to devise an optimal control strategy with adaptation to local circumstances to facilitate the progress towards the elimination of schistosomiasis in Africa and the Philippines [33].

## References

[1] Bergquist R, Utzinger J, Keiser J. 2017 Controlling schistosomiasis with praziquantel: how much longer without a viable alternative? Infectious Diseases of Poverty, 6, 74. 28351414 10.1186/s40249-017-0286-2 PMC5371198

[2] Berry-Cabán CS. 2007 Return of the god of plague: schistosomiasis in China. Journal of Rural and Tropical Public Health, 6, 45–53.

[3] Bundy DA, Gottlieb M. 1999 Parasitic infection in China: farewell to the god of plagues. Parasitology Today, 15, 170–172. 10322345 10.1016/s0169-4758(99)01434-9

[4] Chen MG. 2005 Use of praziquantel for clinical treatment and morbidity control of schistosomiasis japonica in China: a review of 30years’ experience. Acta Tropica, 96, 168–176. 16125657 10.1016/j.actatropica.2005.07.011

[5] Chen MG. 2014 Assessment of morbidity due to *Schistosoma japonicum* infection in China. Infectious Diseases of Poverty, 3, 6. 24529186 10.1186/2049-9957-3-6 PMC3928580

[6] Chen YY, Liu JB, Huang XB, Cai SX, Su ZM, Zhong R, Zou L, Miao XP. 2014 New integrated strategy emphasizing infection source control to curb Schistosomiasis japonica in a marshland area of Hubei Province, China: findings from an eight-year longitudinal survey. PLoS One, 9, e89779. 24587030 10.1371/journal.pone.0089779 PMC3938508

[7] Colley DG. 2014 Morbidity control of schistosomiasis by mass drug administration: how can we do it best and what will it take to move on to elimination? Tropical Medicine and Health, 42, 25–32. 10.2149/tmh.2014-S04 PMC4204048 25425948

[8] Colley DG, Bustinduy AL, Secor WE, King CH. 2014 Human schistosomiasis. Lancet, 383, 2253–2264. 24698483 10.1016/S0140-6736(13)61949-2 PMC4672382

[9] Doenhoff MJ, Hagan P, Cioli D, Southgate V, Pica-Mattoccia L, Botros S, Coles G, TchuemTchuenté LA, Mbaye A, Engels D. 2009 Praziquantel: its use in control of schistosomiasis in sub-Saharan Africa and current research needs. Parasitology, 136, 1825–1835. 19281637 10.1017/S0031182009000493

[10] Engels D, Wang LY, Palmer KL. 2005 Control of schistosomiasis in China. Acta Tropica, 96, 67–68. 16125656 10.1016/j.actatropica.2005.07.004

[11] Gray DJ, Li YS, Williams GM, Zhao ZY, Harn DA, Li SM, Ren MY, Feng Z, Guo FY, Guo JG, Zhou J, Dong YL, Li Y, Ross AG, McManus DP. 2014 A multi-component integrated approach for the elimination of schistosomiasis in the People’s Republic of China: design and baseline results of a 4-year cluster-randomised intervention trial. International Journal for Parasitology, 44, 659–668. 24929133 10.1016/j.ijpara.2014.05.005

[12] Gray DJ, Williams GM, Li Y, McManus DP. 2008 Transmission dynamics of *Schistosoma japonicum* in the lakes and marshlands of China. PLoS One, 3, e4058. 19115007 10.1371/journal.pone.0004058 PMC2605259

[13] Hipgrave D. 2011 Communicable disease control in China: from Mao to now. Journal of Global Health, 1, 224–238. 23198121 PMC3484775

[14] Hong QB, Yang K, Huang YX, Sun LP, Yang GJ, Gao Y, Gao Y, Zhang LH, Zhou M, Steinmann P, Liang YS. 2011 Effectiveness of a comprehensive schistosomiasis japonica control program in Jiangsu province, China, from 2005 to 2008. Acta Tropica, 120, S151–S157. 21147056 10.1016/j.actatropica.2010.11.006

[15] Hong XC, Xu XJ, Chen X, Li YS, Yu CH, Yuan Y, Chen YY, Li RD, Qiu J, Liu ZC, Yi P, Ren GH, He HB. 2013 Assessing the effect of an integrated control strategy for schistosomiasis japonica emphasizing bovines in a marshland area of Hubei Province, China: a cluster randomized trial. PLoS Neglected Tropical Diseases, 7, e2122. 23516656 10.1371/journal.pntd.0002122 PMC3597472

[16] Hotez PJ, Kamath A. 2009 Neglected tropical diseases in sub-saharan Africa: review of their prevalence, distribution, and disease burden. PLoS Neglected Tropical Diseases, 3, e412. 19707588 10.1371/journal.pntd.0000412 PMC2727001

[17] Inobaya MT, Olveda RM, Tallo V, McManus DP, Williams GM, Harn DA, Li Y, Chau TN, Olveda DU, Ross AG. 2015 Schistosomiasis mass drug administration in the Philippines: lessons learnt and the global implications. Microbes and Infection, 17, 6–15. 25448635 10.1016/j.micinf.2014.10.006

[18] King CH. 2010 Parasites and poverty: the case of schistosomiasi. Acta Tropica, 113, 95–104. 19962954 10.1016/j.actatropica.2009.11.012 PMC2812649

[19] King CH. 2017 The evolving schistosomiasis agenda 2007–2017 – Why we are moving beyond morbidity control toward elimination of transmission. PLoS Neglected Tropical Diseases, 11, e0005517. 28426653 10.1371/journal.pntd.0005517 PMC5398522

[20] Li SZ, Qian YJ, Yang K, Wang Q, Zhang HM, Liu J, Chen MH, Huang XB, Xu YL, Bergquist R, Zhou XN. 2012 Successful outcome of an integrated strategy for the reduction of schistosomiasis transmission in an endemically complex area. Geospatial Health, 6, 215–220. 22639123 10.4081/gh.2012.139

[21] Liang S, Yang C, Zhong B, Qiu D. 2006 Re-emerging schistosomiasis in hilly and mountainous areas of Sichuan, China. Bulletin of the World Health Organization, 84, 139–144. 16501732 10.2471/blt.05.025031 PMC2626530

[22] Liu J, Zhu C, Shi Y, Li H, Wang L, Qin S, Kang S, Huang Y, Jin Y, Lin J. 2012 Surveillance of *Schistosoma japonicum* infection in domestic ruminants in the Dongting Lake region, Hunan province, China. PLoS One, 7, e31876. 22359638 10.1371/journal.pone.0031876 PMC3281023

[23] Liu R, Dong HF, Guo Y, Zhao QP, Jiang MS. 2011 Efficacy of praziquantel and artemisinin derivatives for the treatment and prevention of human schistosomiasis: a systematic review and meta-analysis. Parasites and Vectors, 4, 201. 22004571 10.1186/1756-3305-4-201 PMC3207908

[24] Liu R, Dong HF, Jiang MS. 2013 The new national integrated strategy emphasizing infection sources control for schistosomiasis control in China has made remarkable achievements. Parasitology Research, 112, 1483–1491. 23354940 10.1007/s00436-013-3295-5

[25] Liu W, Cao CL, Chen Z, Li SZ, Tang L, Xiao Y, Zhang HM, Yang ZQ, Wang Y, Su SY, Wang LY, Wang Q, Xu JF, Bao ZP, Huang XB, Zhou XN. 2013 Evaluation of the comprehensive schistosomiasis control measures with emphasis on infection source of replacing cattle with machine. Chinese Journal of Parasitology and Parasitic Diseases, 31, 296–301. 24812859

[26] Liu Y, Zhong B, Wu ZS, Liang S, Qiu DC, Ma X. 2017 Interruption of schistosomiasis transmission in mountainous and hilly regions with an integrated strategy: a longitudinal case study in Sichuan, China. Infectious Diseases of Poverty, 6, 79. 28385163 10.1186/s40249-017-0290-6 PMC5383976

[27] Luo TP, Zhou XN, Qiu ZL. 2009 Cost-effectiveness and cost-benefit of integrated schistosomiasis control strategy with emphasis on infectious source control in mountainous areas of Yunnan Province. Chinese Journal of Schistosomiasis Control, 21, 93–97.

[28] Qing-Wu J, Li-Ying W, Jia-Gang G, Ming-Gang C, Xiao-Nong Z, Engels D. 2002 Morbidity control of schistosomiasis in China. Acta Tropica, 82, 115–125. 12020884 10.1016/s0001-706x(02)00006-2

[29] Rollinson D, Knopp S, Levitz S, Stothard JR, TchuemTchuenté LA, Garba A, Mohammed KA, Schur N, Person B, Colley DG, Utzinger J. 2013 Time to set the agenda for schistosomiasis elimination. Acta Tropica, 128, 423–440. 22580511 10.1016/j.actatropica.2012.04.013

[30] Ross AG, Chau TN, Inobaya MT, Olveda RM, Li Y, Harn DA. 2017 A new global strategy for the elimination of schistosomiasis. International Journal of Infectious Diseases, 54, 130–137. 27939558 10.1016/j.ijid.2016.09.023

[31] Ross AG, Olveda RM, Acosta L, Harn DA, Chy D, Li Y, Gray DJ, Gordon CA, McManus DP, Williams GM. 2013 Road to the elimination of schistosomiasis from Asia: the journey is far from over. Microbes and Infection, 15, 858–865. 23973709 10.1016/j.micinf.2013.07.010 PMC4433715

[32] Ross AG, Olveda RM, Chy D, Olveda DU, Li Y, Harn DA, Gray DJ, McManus DP, Tallo V, Chau TN, Williams GM. 2015 Can mass drug administration lead to the sustainable control of schistosomiasis? Journal of Infectious Diseases, 211, 283–289. 25070942 10.1093/infdis/jiu416

[33] Savioli L, Albonico M, Colley DG, Correa-Oliveira R, Fenwick A, Green W, Kabatereine N, Kabore A, Katz N, Klohe K, LoVerde PT, Rollinson D, Stothard JR, Tchuem Tchuenté LA, Waltz J, Zhou XN. 2017 Building a global schistosomiasis alliance: an opportunity to join forces to fight inequality and rural poverty. Infectious Diseases of Poverty, 6, 65. 28330495 10.1186/s40249-017-0280-8 PMC5363045

[34] Schwarzer G. 2013 meta: Meta-Analysis with R.R package version 3.0-1. http://CRAN.R-project.org/package=meta.

[35] Sesay S, Paye J, Bah MS, McCarthy FM, Conteh A, Sonnie M, Hodges MH, Zhang Y. 2014 *Schistosoma mansoni* infection after three years of mass drug administration in Sierra Leone. Parasites and Vectors, 7, 14. 24401567 10.1186/1756-3305-7-14 PMC3895768

[36] Seto EY, Remais JV, Carlton EJ, Wang S, Liang S, Brindley PJ, Qiu D, Spear RC, Wang LD, Wang TP, Chen HG, Dong XQ, Wang LY, Hao Y, Bergquist R, Zhou XN. 2011 Toward sustainable and comprehensive control of schistosomiasis in China: lessons from Sichuan. PLoS Neglected Tropical Diseases, 5, e1372. 22039563 10.1371/journal.pntd.0001372 PMC3201916

[37] Steinmann P, Keiser J, Bos R, Tanner M, Utzinger J. 2006 Schistosomiasis and water resources development: systematic review, meta-analysis, and estimates of people at risk. Lancet Infectious Diseases, 6, 411–425. 16790382 10.1016/S1473-3099(06)70521-7

[38] Sterne JA, Sutton AJ, Ioannidis JP, Terrin N, Jones DR, Lau J, Carpenter J, Rücker G, Harbord RM, Schmid CH, Tetzlaff J, Deeks JJ, Peters J, Macaskill P, Schwarzer G, Duval S, Altman DG, Moher D, Higgins JP. 2011 Recommendations for examining and interpreting funnel plot asymmetry in meta-analyses of randomised controlled trials. British Medical Journal, 343, d4002. 21784880 10.1136/bmj.d4002

[39] Sun LP, Wang W, Liang YS, Tian ZX, Hong QB, Yang K, Yang GJ, Dai JR, Gao Y. 2011 Effect of an integrated control strategy for schistosomiasis japonica in the lower reaches of the Yangtze River, China: an evaluation from 2005 to 2008. Parasites and Vectors, 4, 243. 22208620 10.1186/1756-3305-4-243 PMC3285052

[40] Utzinger J, Zhou XN, Chen MG, Bergquist R. 2005 Conquering schistosomiasis in China: the long march. Acta Tropica, 96, 69–96. 16312039 10.1016/j.actatropica.2005.08.004

[41] Viechtbauer W. 2010 Conducting meta-analyses in R with the metafor package. Journal of Statistical Software, 36, 1–48.

[42] Wang LD, Chen HG, Guo JG, Zeng XJ, Hong XL, Xiong JJ, Wu XH, Wang XH, Wang LY, Xia G, Hao Y, Chin DP, Zhou XN. 2009 A strategy to control transmission of *Schistosoma japonicum* in China. New England Journal of Medicine, 360, 121–128. 19129526 10.1056/NEJMoa0800135

[43] Wang LD, Guo JG, Wu XH, Chen HG, Wang TP, Zhu SP, Zhang ZH, Steinmann P, Yang GJ, Wang SP, Wu ZD, Wang LY, Hao Y, Bergquist R, Utzinger J, Zhou XN. 2009 China’s new strategy to block *Schistosoma japonicum* transmission: experiences and impact beyond schistosomiasis. Tropical Medicine and International Health, 14, 1475–1483. 19793080 10.1111/j.1365-3156.2009.02403.x

[44] Wang LD, Utzinger J, Zhou XN. 2008 Schistosomiasis control: experiences and lessons from China. Lancet, 372, 1793–1795. 18930529 10.1016/S0140-6736(08)61358-6 PMC7135384

[45] Wang W, Dai JR, Liang YS. 2014 Apropos: factors impacting on progress towards elimination of transmission of schistosomiasis japonica in China. Parasites and Vectors, 7, 408. 25175021 10.1186/1756-3305-7-408 PMC4261779

[46] Wang X, Wang W, Wang P. 2017 Long-term effectiveness of the integrated schistosomiasis control strategy with emphasis on infectious source control in China: a 10-year evaluation from 2005 to 2014. Parasitology Research, 116, 521–528. 27812902 10.1007/s00436-016-5315-8

[47] Wu W, Wang W, Huang YX. 2011 New insight into praziquantel against various developmental stages of schistosomes. Parasitology Research, 109, 1501–1507. 21984370 10.1007/s00436-011-2670-3

[48] Wu XH, Chen MG, Zheng J. 2005 Surveillance of schistosomiasis in five provinces of China which have reached the national criteria for elimination of the disease. Acta Tropica, 96, 276–281. 16198301 10.1016/j.actatropica.2005.07.021

[49] Wu XH, Zhang SQ, Xu XJ, Huang YX, Steinmann P, Utzinger J, Wang TP, Xu J, Zheng J, Zhou XN. 2008 Effect of floods on the transmission of schistosomiasis in the Yangtze River valley, People’s Republic of China. Parasitology International, 57, 271–276. 18499513 10.1016/j.parint.2008.04.004

[50] Xianyi C, Liying W, Jiming C, Xiaonong Z, Jiang Z, Jiagang G, Xiaohua W, Engels D, Minggang C. 2005 Schistosomiasis control in China: the impact of a 10-year World Bank Loan Project (1992–2001). Bulletin of the World Health Organization, 83, 43–48. 15682248 PMC2623468

[51] Xiao SH, Keiser J, Chen MG, Tanner M, Utzinger J. 2010 Research and development of antischistosomal drugs in the People’s Republic of China a 60-year review. Advances in Parasitology, 73, 231–295. 20627145 10.1016/S0065-308X(10)73009-8

[52] Xu J, Bergquist R, Qian YJ, Wang Q, Yu Q, Peeling R, Croft S, Guo JG, Zhou XN. 2016 China-Africa and China-Asia Collaboration on schistosomiasis control: a SWOT analysis. Advances in Parasitology, 92, 435–466. 27137455 10.1016/bs.apar.2016.02.005

[53] Xu J, Steinman P, Maybe D, Zhou XN, Lv S, Li SZ, Peeling R. 2016 Evolution of the National Schistosomiasis Control Programmes in the People’s Republic of China. Advances in Parasitology, 92, 1–38. 27137441 10.1016/bs.apar.2016.02.001

[54] Xu J, Xu JF, Li SZ, Zhang LJ, Wang Q, Zhu HH, Zhou XN. 2015 Integrated control programmes for schistosomiasis and other helminth infections in P.R. China. Acta Tropica, 141, 332–341. 24361182 10.1016/j.actatropica.2013.11.028

[55] Xu J, Yu Q, Tchuenté LA, Bergquist R, Sacko M, Utzinger J, Lin DD, Yang K, Zhang LJ, Wang Q, Li SZ, Guo JG, Zhou XN. 2016 Enhancing collaboration between China and African countries for schistosomiasis control. Lancet Infectious Diseases, 16, 376–383. 26851829 10.1016/S1473-3099(15)00360-6

[56] Yang GJ, Liu L, Zhu HR, Griffiths SM, Tanner M, Bergquist R, Utzinger J, Zhou XN. 2014 China’s sustained drive to eliminate neglected tropical diseases. Lancet Infectious Diseases, 14, 881–892. 24875936 10.1016/S1473-3099(14)70727-3

[57] Yang K, Li Hong J, Yang WC, Shi XW, Qi YL. 2009 Effect of comprehensive schistosomiasis control measures with emphasis on infectious source control in dam areas of mountainous region, Yunnan Province. Chinese Journal of Schistosomiasis Control, 21, 272–275.

[58] Yang Y, Zhou YB, Song XX, Li SZ, Zhong B, Wang TP, Bergquist R, Zhou XN, Jiang QW. 2016 Integrated control strategy of schistosomiasis in the People’s Republic of China: projects involving agriculture, water conservancy, forestry, sanitation and environmental modification. Advances in Parasitology, 92, 237–268. 27137449 10.1016/bs.apar.2016.02.004

[59] Yu LQ, Qiu XY, Hu Y, Wang YL. 2010 Effect of replacing bovine with tractors for farming on schistosomiasis control in Xuancheng City. Chinese Journal of Schistosomiasis Control, 22, I–II

[60] Yu Q, Zhao GM, Hong XL, Lutz EA, Guo JG. 2013 Impact and cost-effectiveness of a comprehensive schistosomiasis japonica control program in the Poyang Lake region of China. International Journal of Environmental Research and Public Health, 10, 6409–6421. 24287861 10.3390/ijerph10126409 PMC3881122

[61] Zhang SQ, Wang TP, Tao CG, Chen GX, Chen JS, Xu H, Yin NW, Wang H, Ge JH. 2005 Observation on comprehensive measures of safe treatment of night-soil and water supply, replacement of bovine with machine for schistosomiasis control. Chinese Journal of Schistosomiasis Control, 17, 437–442.

[62] Zhou XN, Bergquist R, Leonardo L, Yang GJ, Yang K, Sudomo M, Olveda R. 2010 Schistosomiasis japonica control and research needs. Advances in Parasitology, 72, 145–148. 20624531 10.1016/S0065-308X(10)72006-6

[63] Zhou XN, Guo JG, Wu XH, Jiang QW, Zheng J, Dang H, Wang XH, Xu J, Zhu HQ, Wu GL, Li YS, Xu XJ, Chen HG, Wang TP, Zhu YC, Qiu DC, Dong XQ, Zhao GM, Zhang SJ, Zhao NQ, Xia G, Wang LY, Zhang SQ, Lin DD, Chen MG, Hao Y. 2007 Epidemiology of schistosomiasis in the People’s Republic of China, 2004. Emerging Infectious Diseases, 13, 1470–1476. 18257989 10.3201/eid1310.061423 PMC2851518

[64] Zhou XN, Wang LY, Chen MG, Wu XH, Jiang QW, Chen XY, Zheng J, Utzinger J. 2005 The public health significance and control of schistosomiasis in China – then and now. Acta Tropica, 96, 97–105. 16125655 10.1016/j.actatropica.2005.07.005

[65] Zhou YB, Liang S, Chen GX, Rea C, He ZG, Zhang ZJ, Wei JG, Zhao GM, Jiang QW. 2011 An integrated strategy for transmission control of *Schistosoma japonicum* in a marshland area of China: findings from a five-year longitudinal survey and mathematical modeling. American Journal of Tropical Medicine and Hygiene, 85, 83–88. 21734130 10.4269/ajtmh.2011.10-0574 PMC3122349

[66] Zhou YB, Liang S, Chen GX, Rea C, Han SM, He ZG, Li YP, Wei JG, Zhao GM, Jiang QW. 2013 Spatial-temporal variations of *Schistosoma japonicum* distribution after an integrated national control strategy: a cohort in a marshland area of China. BMC Public Health, 13, 297. 23556428 10.1186/1471-2458-13-297 PMC3621803

[67] Zou L, Ruan S. 2015 Schistosomiasis transmission and control in China. Acta Tropica, 143, 51–57. 25559046 10.1016/j.actatropica.2014.12.004

